# Usage of intermingled skin allografts and autografts in a senior patient with major burn injury

**DOI:** 10.1515/med-2021-0395

**Published:** 2021-11-15

**Authors:** Hongmin Luo, Huining Bian, Chuanwei Sun, Shaoyi Zheng, Bing Xiong, Zhifeng Huang, Zuan Liu, Lianghua Ma, Hanhua Li, Huade Chen, Wen Lai

**Affiliations:** Department of Burns and Wound Repair Surgery, Guangdong Provincial People’s Hospital, Guangdong Academy of Medical Sciences, Guangzhou 510080, China

**Keywords:** major burn injury, senior patients, intermingled skin allografts and autografts

## Abstract

Mortality rate in older adults following extensive burn injury is extremely high, and management of these patients is challenging. One of the main problems is that autologous split-thickness skin grafts are scarce and the wounds cannot be covered quickly and effectively. Intermingled skin grafting is a low-tech and economic method, which not only maximizes the use of precious autologous skin but also prevents the wounds from infection and consumption. Herein we present a case of extensive burn injury in a 68-year-old female successfully treated with intermingled skin grafting. The patient was accidentally burned by gas flame, resulting in a major burn injury covering 80% of her total body surface area. Early burn wound excision was performed and the wound was temporarily covered with irradiated porcine skin in the first week after injury. Autologous stamp-like skin grafts were applied to the wound bed 4 weeks after injury. In this operation, the results were not satisfactory. The take rate of the skin grafts is only about 50%. We covered the wounds with intermingled skin allografts and autografts 8 weeks after injury: autografts (0.5 cm × 0.5 cm) + fresh close relative’s allografts (1 cm × 1 cm) + cryopreserved allografts (2 cm × 2 cm).

## 1 Introduction

The problem of aging is becoming more and more serious, and there is likely to be an increasing number of senior patients suffering from burn injury each year. Many elderly people have pre-existing medical conditions and aging-associated decline in organ function. Therefore, the mortality rate in older adults after major burn injury is extremely high, and management of these patients is challenging [1,2]. In addition, older adults after burn injury are likely to have poor functional recovery [3]. Extensive burn injury can result in loss of skin barrier function which leads to fluid loss and infection. The elderly people are obviously more sensitive to these harmful changes. If the burned wounds cannot be covered effectively and on time, these patients will inevitably die from infection and consumption. Therefore, it is critical to cover the wounds quickly and effectively. Autologous skin grafting is the only way to permanently cover the wounds. However, it is obviously unrealistic to completely cover all the wounds with autogenous skin grafts in the early stage as these patients have little donor skin. Another low-tech and economic alternative is usage of intermingled skin allografts and autografts. This method not only maximizes the use of precious donor skin but also effectively covers the wounds to avoid infection and consumption caused by wound exposure. Herein we present a case of extensive burn injury in a 68-year-old female successfully treated with intermingled skin allografts and autografts.

## 2 Case presentation

**Figure 1:**
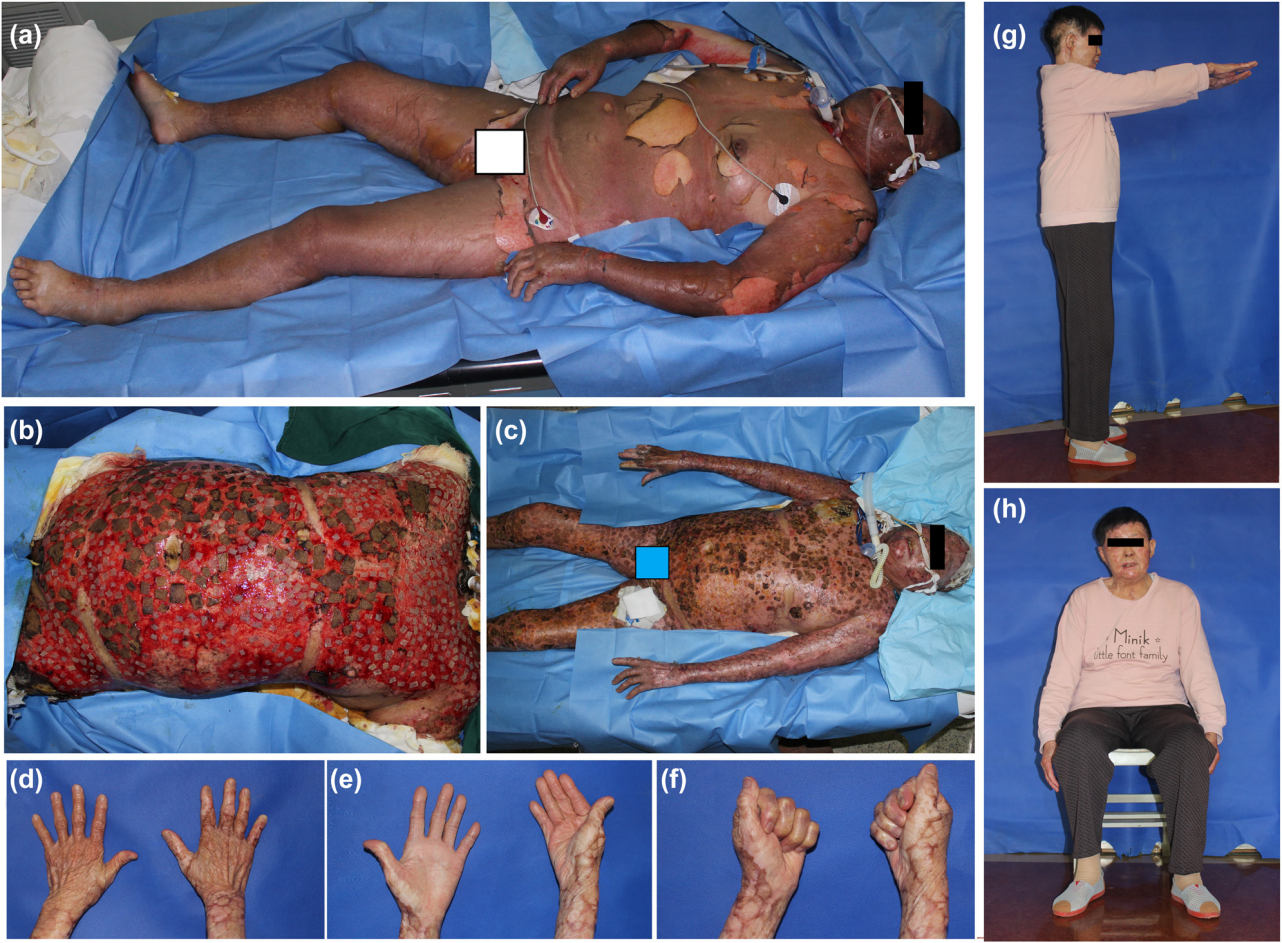
Usage of intermingled skin allografts and autografts in a senior patient with major burn injury. (a) The patient was accidentally burned by gas flame, resulting in a major burn injury covering 80% of her TBSA. Most of the them were third-degree burn wounds. (b) In the eighth week after the injury, the wounds were covered with intermingled skin allografts and autografts. (c) The autografts and fresh allografts expanded and the cryopreserved allografts necrotized and fed off gradually from the edge to the center. There was no obvious wound exposure during the entire treatment process. (d)–(h) After a year of follow-up, the patient recovered well and regained satisfactory function.


A 68-year-old female was accidentally burned by the gas flame while taking bath on October 10, 2018. She has a history of hypertension, diabetes, and atrial fibrillation. She was transferred from the local hospital to our hospital 46 h after the injury. Tracheotomy was performed on admission and the patient had poor mental response, shortness of breath, and rapid heart rate (160/min). About 80% of the total body surface area (TBSA) was burned, most of them were third-degree burns (Figure 1a). Early burn wound excision was performed and the wounds were temporarily covered with irradiated porcine skin in the first week after injury. Four weeks after the injury, healthy granulation was formed in the wound bed. At this time, we performed a second operation on the patient: autologous stamp-like skin grafting was performed on the upper and lower limbs. In this operation, the results were not satisfactory. The take rate of the skin grafts is only about 50%. In the eighth week after the injury, we performed the third operation. This time we covered the wounds with intermingled skin allografts and autografts (Figure 1b): small skin autografts (0.5 cm × 0.5 cm) + moderate close relative’s fresh allografts (1 cm × 1 cm) + large cryopreserved allografts (2 cm × 2 cm). One son of the patient donated his scalp skin, and HIV and hepatitis were excluded preoperatively. A Zimmer electric-driven dermatome set was used to harvest intermediate split-thickness skin allografts from the scalp of the donor (3% TBSA). Then, the burned wounds of the patient were debrided and split-thickness autografts from scalp (2% TBSA) were harvested. The autografts, close relative’s fresh allografts, and cryopreserved allografts were cut into different sizes: 0.5 cm × 0.5 cm, 1 cm × 1 cm, and 2 cm × 2 cm, respectively. Then, these intermingled skin grafts were applied to the wound bed. Wound dressings were changed every 2–3 days. There were partial loss of the intermingled grafts and cryopreserved allografts were used to avoid development of newly exposed wounds during wound care. The autografts and fresh allografts expanded and the cryopreserved allografts necrotized and fed off gradually from the edge to the center (Figure 1c). We did not see obvious large area rejection of the fresh allografts. Instead, we found some epithelial layer of the fresh allografts fed off but was re-epithelialized by the autografts very soon. And there was no obvious wound exposure during the entire treatment process. At 3 months after the injury, most of the wounds had healed, leaving about 3% of the TBSA wounds on the back region. At this time, we performed one more operation for the patient: covering the wounds with autologous stamp-like skin grafts. The patient was discharged from the hospital 4 months after the injury. After a year of follow-up, the patient recovered well and regained satisfactory function (Figure 1d–h).

**Ethics approval and consent to participate:** The institute ethics committee approval is waived for retrospective analysis of clinical records. Consent to participate was obtained.
**Consent for publication:** The submission contains clinical photographs of the patient and consent to publish the photographs has been obtained.

## 3 Discussion

Burns are an important cause of morbidity and mortality worldwide, especially among the elderly. With the progress of the aging society, the elderly population is growing rapidly, and the incidence of burns is likely to increase year by year. Compared with younger adults, older adults are less likely to tolerate major burn injury and have a lower chance of survival. The patient in this report is a 68-year-old female with a 80% TBSA burn injury, and her survival possibilities were extremely low according to our experience. The key to the success of the treatment was whether the necrotic tissue can be removed as soon as possible and the wound can be effectively covered to prevent infection and consumption. Early tangential excision was performed 1 week after injury. At this time, the patient had been adequately resuscitated and large scale of burn toxin absorption did not take place, so it was a relatively safe timing. The first tangential excision was relatively conservative and most of the degenerated dermis were retained. At this time, the “take” rate of autologous skin grafting was very low, so we chose to apply irradiated porcine skin to temporarily cover the wound bed. In the fourth week after the injury, healthy granulation was formed in the wound bed and we covered the wounds of the limbs with autologous stamp-like skin grafts. In this operation, the results were not satisfactory. The take rate of the skin grafts is only about 50%. The reason for the failure of the operation may be due to the poor self-growth ability of the patient. Another reason was that there was no effective wound covering between the stamp-like skin grafts, making it very easy to get infected. Therefore, covering the wounds effectively was critical. There are several reports using close relative intermingled skin allografts and autografts in the treatment of major burns in children [4,5]. The advantage of this method for children is that only a small amount of allografts is needed to cover most of the wounds. In our case, covering the wounds completely with a close relative’s intermingled skin allografts and autografts is obviously unrealistic. Therefore, we chose to cover the wounds with small skin autografts, moderate close relative’s fresh allografts, and large cryopreserved allografts. This method has its own advantage because the rejection of close relative’s fresh allografts is relatively mild and the allografts survive longer. In fact, during the entire treatment process, the cryopreserved allografts gradually necrotized and shed off from the surrounding to the center, while the autografts and close relative’s fresh allografts continued to expand. In addition, we did not observe obvious large area rejection of the fresh allografts. Instead, we found some epithelial layer of the fresh allografts fed off but was re-epithelialized by the autografts soon. It is a coherent process and there is no obvious wound exposure during the whole treatment process. In conclusion, for older adults with extensive burns, it is an effective method to cover the wounds with small skin autografts, moderate close relative’s fresh allografts, and large cryopreserved allografts. This method prevents the patient from obvious wound exposure during the entire treatment process, which not only maximizes the use of precious autologous skin but also effectively covers the wounds to avoid infection and consumption caused by wound exposure.
